# OMP38 of Carbapenem‐Resistant *Acinetobacter Baumannii*‐Mediated mtDNA Release Activates the cGAS‐STING Signaling to Induce Inflammatory Response

**DOI:** 10.1002/advs.202408292

**Published:** 2024-12-04

**Authors:** Yang Yang, Yuanyuan Zeng, Jianjie Zhu, Jianjun Li, Lei Gu, Lin Wei, Zeyi Liu, Jian‐an Huang

**Affiliations:** ^1^ Department of Pulmonary and Critical Care Medicine the First Affiliated Hospital of Soochow University Suzhou 215006 China; ^2^ Institute of Respiratory Diseases Soochow University Suzhou 215006 China; ^3^ Suzhou Key Laboratory for Respiratory Diseases Suzhou 215006 China; ^4^ Department of Infectious Diseases The First Affiliated Hospital of Anhui Medical University Hefei Anhui 230032 China; ^5^ School of Life Sciences Anhui Medical University Hefei Anhui 230032 China

**Keywords:** *Acinetobacter baumannii*, carbapenem‐resistant, cGAS‐STING, mitochondrial DNA, outer membrane protein 38

## Abstract

Carbapenem‐resistant *Acinetobacter baumannii* (CRAB) has become a major threat in the treatment of bacterial infection, and immunotherapy in a non‐antibiotic‐dependent manner is an effective way to overcome CRAB infection. However, the role of the innate immune response in CRAB infection is poorly understood. Here, it is reported that CRAB infection induced a cytosolic DNA‐sensing signaling pathway and significant IFN‐β production in mice post‐CRAB infection. The knockout of STING reduced bacterial burden, the production of inflammatory cytokines, and lung injury in mice post CRAB infection. The cytosolic DNA sensor cyclic GMP‐AMP synthase (cGAS) and the adaptor protein stimulator of interferon genes (STING) are required for CRAB‐induced IFN‐β expression in macrophages. Intriguingly, CRAB utilized outer membrane vesicles (OMVs) to transport outer membrane protein 38 (OMP38) into mitochondria, triggering mitochondrial DNA (mtDNA) release into the cytosol through the mitochondrial permeability transition pore (mPTP) and activating the cGAS‐STING signaling. Finally, epigallocatechin gallate (EGCG) is demonstrated to block the activation of the cGAS‐STING pathway and ameliorate CRAB‐induced excessive inflammatory response. These results demonstrated that the early innate immune response to CRAB infection is activated in a cGAS‐STING‐dependent manner, which could be a potential therapeutic target for CRAB infection.

## Graphical abstract

In article, Lin Wei, Zeyi Liu, Jian‐an Huang and co‐workers reveal a new way to combat carbapenem‐resistant *Acinetobacter baumannii* (CRAB). CRAB uses OMVs to transport virulence factor OMP38, triggering mitochondrial DNA release and activating the cGAS‐STING pathway for inflammation. Epigallocatechin gallate (EGCG) blocks this pathway, suggesting a new strategy against antibiotic resistance.

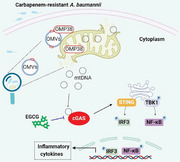

## 1 Introduction

Carbapenem‐resistant *Acinetobacter baumannii* (CRAB) has emerged as a prominent cause of nosocomial infections, with a rising impact on critically ill individuals, yet no potential drugs have been identified in clinical studies to consistently reduce mortality risk or improve patient outcomes.^[^ ^1^ ^]^ Indeed, CRAB has been listed by the World Health Organization (WHO) as one of the three top‐priority pathogens (Priority 1: Critical) for which the discovery and development of novel antibiotics are urgently needed.^[^ ^2^ ^]^ Globally, the proportion of CRAB varies from 30% to 80%, with the highest proportion in Asia.^[^ ^3^, ^4^ ^]^ With the high mortality rates associated with CRAB infection, the lack of antimicrobial agents in the development pipeline, and the common association between carbapenem resistance and genes encoding oxacillinase (OXA) carbapenemases, such as OXA‐23 and OXA‐24/40, the development of novel antimicrobial agents and alternative therapeutic approaches are critically needed.^[^ ^5^ ^]^

The host's innate immune system serves as the first line of host defence and recognizes *A. baumannii* within hours of infection, which determines the outcome of the infection.^[^ ^6^ ^]^ The recruitment and activation of innate immune cells are key features of host defence against *A. baumannii* infection, and alveolar macrophages have been found to play a significant role in *A. baumannii* clearance.^[^ ^7^ ^]^ Although few studies have focused on the interaction between CRAB and the innate immune system, the innate immune response may provide potential therapeutic targets for developing new strategies to combat CRAB infection.

The innate immune system uses pattern recognition receptors (PRRs) to detect pathogen‐associated molecular patterns (PAMPs) (e.g., lipopolysaccharide (LPS), and capsules) and damage‐associated molecular patterns (DAMPs) (host cell components released during infection‐induced cell damage or death).^[^ ^8^ ^]^ The combined findings from polymorphism analysis in patients, in vitro human and murine cell culture, and animal models have validated TLR4 as the primary PRR for the initial interaction between *A. baumannii* and host cells.^[^ ^9^ ^]^ However, research suggests that TLR2 plays distinct roles in the host‐*A. baumannii* interaction. TLR function is influenced by various parameters, including infection route, stage, and severity. Intracellular immune sensing and signaling molecules such as TLR9 and NOD2 play crucial roles in the immune response to *A. baumannii*.^[^ ^10^, ^11^ ^]^ However, the mechanism by which these PRRs respond to CRAB needs further study.

Cytosolic dsDNA sensing, mediated by the cyclic‐GMP‐AMP (cGAMP) synthase (cGAS)‐stimulator of interferon genes (STING) pathway, has recently been considered an essential component for linking dsDNA (originating from DNA viruses, reverse‐transcription of RNA viruses, bacteria, parasites, mitochondria, and extracellular vesicles) detection to the initiation of the host's innate immune defence response.^[^ ^12^ ^]^ Increasing research has shown that the cGAS‐STING pathway is critical for inducing a type I IFN response upon infection by a variety of intracellular bacterial pathogens. cGAS detects the bacterial dsDNA of *Mycobacterium tuberculosis*,^[^ ^13^ ^]^ *Chlamydia trachomatis*,^[^ ^14^ ^]^ *Francisella*,^[^ ^15^ ^]^ *Pseudomonas aeruginosa*,^[^ ^16^ ^]^ and *Listeria monocytogenes*.^[^ ^17^ ^]^ In addition, mitochondrial DNA release triggers the cGAS‐STING pathway to induce a type I interferon response during *Salmonella typhimurium* ^[^ ^18^ ^]^ and *Pseudomonas aeruginosa* ^[^ ^19^ ^]^ infections. Whether the cGAS‐STING pathway contributes to the CRAB‐induced host innate immune response is still unknown.

In this study, we explored the role of the cGAS‐STING pathway during CRAB infection by performing transcriptomic analysis and using STING knockout mice. Our results revealed that CRAB activated the cytosolic DNA‐sensing pathway and type I IFN response, which was suppressed by cGAS and STING deficiency. Interestingly, OMP38 of CRAB was localized to the mitochondria via OMVs and the cGAS‐STING pathway was activated by CRAB‐induced mitochondrial DNA release through the mitochondrial permeability transition pore (mPTP). Furthermore, epigallocatechin gallate (EGCG) specifically blocked cGAS activation to support the host defence against CRAB infection. In conclusion, we demonstrated that CRAB triggers mtDNA release to induce cGAS‐STING pathway activation, resulting in inflammatory injury to the lung. This is the first investigation of the role of the cGAS‐STING pathway in CRAB infection, providing a new perspective on the innate immune response to CRAB and a potential therapeutic strategy for treating CRAB infection.

## 2 Results

### 2.1 CRAB Infection Induces Type I IFN Response

**Figure 1:**
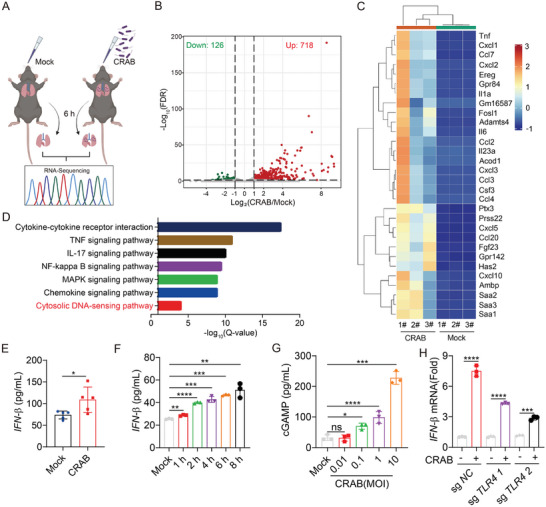
CRAB infection induces a type I IFN response in mice. A) Schematic diagram of the experiment in which mice were left uninfected or infected with 1 × 10^7^ CFU of CRAB for 6 h (*n* = 3). B) Volcano plot showing gene expression analysis of lung tissues; each group had three biological replicates. Differentially expressed genes were those with a false discovery rate (FDR) cut‐off of 0.05 and a fold change of ≥±2. C) Heatmap of the RNA‐seq data. D) Ingenuity pathway analysis of upregulated gene expression changes. E) ELISA analysis of IFN‐β production in lung tissues of uninfected mice or mice infected with 1 × 10^7^ CFU CRAB for 6 h (*n* = 5). F) ELISA analysis of IFN‐β production in mouse peritoneal macrophages uninfected or infected with CRAB for 1, 2, 4, 6, or 8 h at an MOI of 10 (*n* = 3). G) ELISA analysis of cGAMP in mouse peritoneal macrophages uninfected or infected with CRAB for 2 h at an MOI of 0.01, 0.1, 1, 10 (*n* = 3). H) qRT‐PCR analysis of IFN‐β expression in TLR4 knockout iBMDMs generated by CRISPR‐Cas9 and treated with CRAB for 4 h at an MOI of 10 (*n* = 3). The data are expressed as the mean ± SD; *p < 0.05, **p < 0.01, ***p < 0.001, ****p < 0.0001.

Macrophages initiate a strong innate immune response to control bacterial infection by recognizing pathogen‐associated molecular patterns (PAMPs) through pattern recognition receptors (PRRSs) in the early stage of infection. To explore the role of the innate immune response during CRAB infection, mice were infected with 1 × 10^7^ CFU of CRAB through intranasal inoculation. At 6 h post‐infection, lung tissues were collected for high‐throughput RNA sequencing. Compared to the uninfected mice, 844 genes were found to be differentially expressed (≥2‐fold) with a false discovery rate (FDR) cut‐off of 0.05. Among them, 718 genes were upregulated, and 126 genes were downregulated (**Figure** **1**A,B). To validate the RNA‐seq results, the upregulated genes, including TNF‐α, IL‐6, IL‐1β, and CXCL10, were further confirmed by qRT‐PCR (Figure S1A, Supporting Information). In particular, series of pro‐inflammatory cytokines and chemokines were upregulated in the lung of CRAB‐infected mice (Figure 1C).

KEGG pathway enrichment analysis of the differentially expressed genes (DEGs) revealed that the upregulated genes were involved in cytokine‒cytokine receptor interaction, the NF‐κB signaling pathway, the MAPK signaling pathway, and the cytosolic DNA‐sensing pathway (Figure 1D). The involvement of cytosolic DNA‐sensing pathway indicated the activation of the IFN pathway post‐CRAB infection. As expected, IFN‐β was significantly increased in the lung of CRAB‐infected mice and mainly observed in lung macrophages (Figure 1E; Figure S1B, Supporting Information). Furthermore, IFN‐β and cGAMP was obviously up‐regulated in mouse macrophages upon CRAB stimulation (Figure 1F,G; Figure S1C, Supporting Information). It has been reported that *A. baumannii* induced IFN response via a surface innate immune receptor (TLR4).^[^ ^20^ ^]^ However, it is worthy to note that a slight but statistically significant upregulated the expression of IFN‐β was detected in CRAB‐infected TLR4 knockout macrophages (Figure 1H; Figure S1D, Supporting Information). Overall, we demonstrated that CRAB activated the type I IFN response in a TLR4‐independent manner.

### 2.2 STING Knockout Inhibits CRAB Infection in Mice

**Figure 2:**
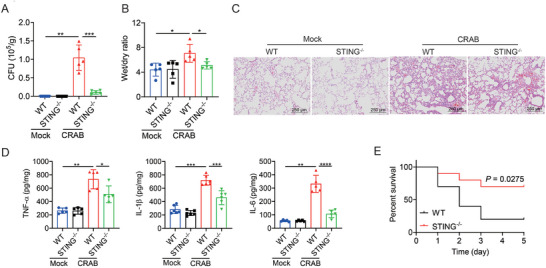
Activation of the cGAS‐STING pathway is associated with CRAB‐induced lung injury in mice. A) WT and STING^−/−^ mice were uninfected or infected with 1 × 10^7^ CFU of CRAB for 24 h (*n* = 5). Lung tissues homogenates were plated to determine the bacterial CFU per gram. B) Wet weight and dry weight of lung tissues were determined (*n* = 5). C) Pathological histological analysis of lung sections stained with H&E. Scale bar: 250 µm. D) ELISA analysis of TNF‐α, IL‐1β, and IL‐6 cytokine levels in lung tissues (*n* = 5). E) WT and STING^−/−^ mice were left uninfected or infected with 1 × 10^8^ CFU of CRAB (*n* = 10). The survival rate of the mice was monitored for up to 5 days. The data are expressed as the mean ± SD; *p < 0.05, **p < 0.01, ***p < 0.001, ****p < 0.0001.

To confirm the role of the cGAS‐STING pathway during CRAB infection, STING knockout (STING^−/−^) mice were infected with CRAB, and the bacterial load of WT and STING^−/‐^ mice were examined at 24 h post‐infection. Compared to WT mice, the bacterial burden, wet/dry ratio of lung, inflammatory injury in lung, and production of pro‐inflammatory cytokines in the lung were significantly decreased in STING^−/−^ mice (**Figure** **2**A–D). Next, WT and STING^−/−^ mice were intragastrically infected with 1×10^8^ CFU of CRAB, and survival rate was monitored. As shown in Figure 2E, STING knockout significantly increased the survival rate of mice post‐CRAB infection as compared to WT mice. Upon CRAB challenge, the survival rate of WT mice was just 20%, while the survival rate was significantly increased to 70% after STING^−/−^ knockout. Taken together, these results indicated that the cGAS‐STING pathway was involved in CRAB infection.

### 2.3 The CRAB‐Induced Inflammatory Response is Associated with Activation of the cGAS‐STING Pathway

**Figure 3:**
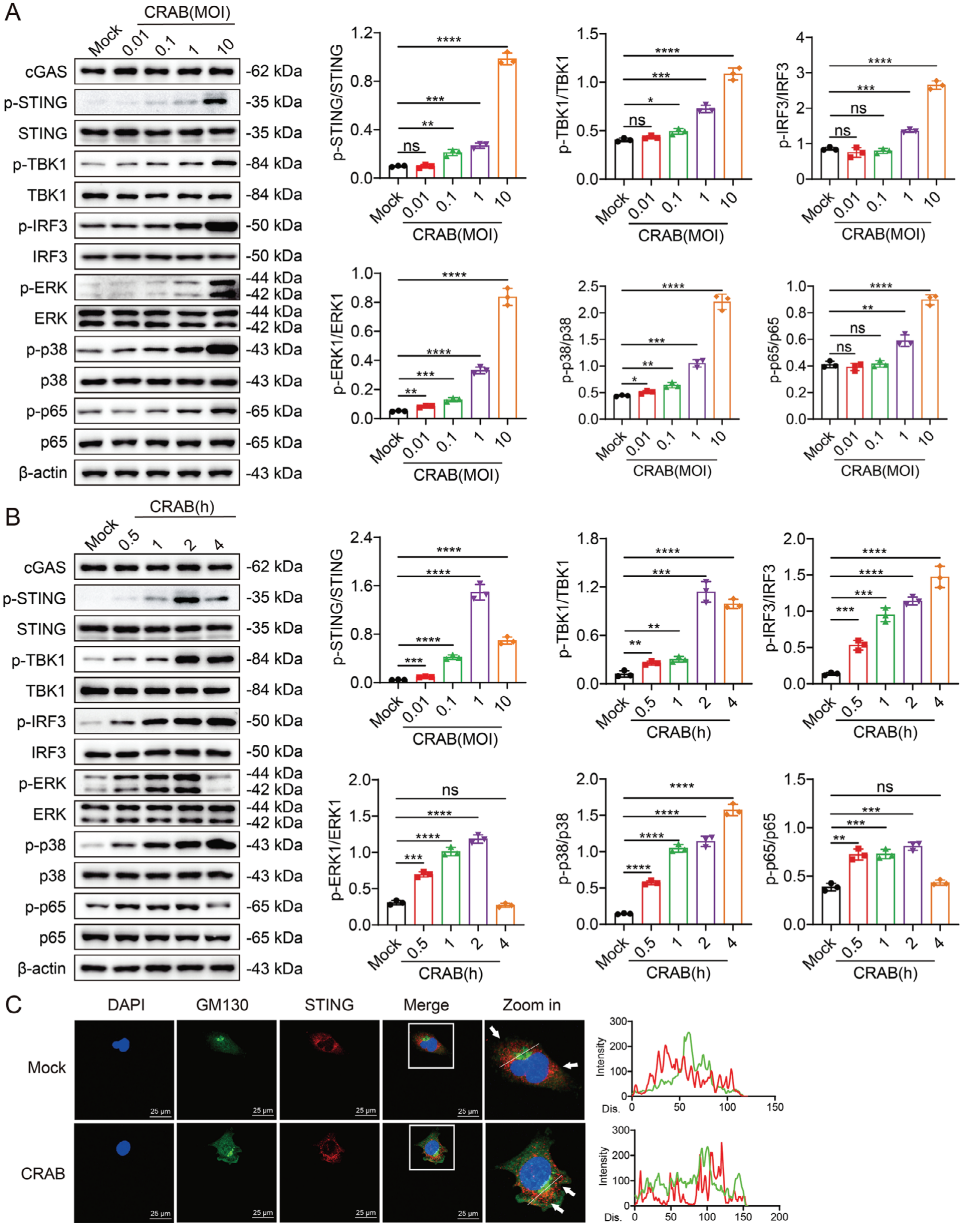
The CRAB‐induced type I IFN response is associated with activation of the cGAS‐STING pathway. A) Immunoblot analysis of protein expression in peritoneal macrophages treated with CRAB for 2 h at MOI of 0.01, 0.1, 1, and 10 (*n* = 3). B) Immunoblot analysis of protein expression in peritoneal macrophages treated with CRAB for 0.5, 1, 2, or 4 h at an MOI of 10 (*n* = 3). C) Immunofluorescence staining analysis of the Golgi apparatus translocation of STING in peritoneal macrophages treated with CRAB for 1 h at an MOI of 10. Scale bar: 25 µm. The data are expressed as the mean ± SD; *p < 0.05, **p < 0.01, ***p < 0.001, ****p < 0.0001.

Innate immune cells recognize *A. baumannii* and initiate the production of pro‐inflammatory cytokines and chemokines, which largely determine the fate of the infection.^[^ ^6^ ^]^ Macrophages, the main immune cell population of the alveolar airspace, are endowed with cytosolic and membrane receptors, and are critical for anti‐infective immune response upon many respiratory bacterial infection.^[^ ^21^ ^]^ Therefore, we further evaluated the role of the cGAS‐STING pathway in CRAB‐infected macrophages. CRAB induced the phosphorylation of STING, TBK1, IRF3, p38, ERK, and NF‐κB p65 in macrophages in a dose‐ and time‐dependent manner. CRAB infection did not affect the expression of cGAS or STING (**Figure** **3**A,B; Figure S2, Supporting Information), but CRAB infection induced the translocation of STING to the Golgi apparatus (Figure 3C).

**Figure 4:**
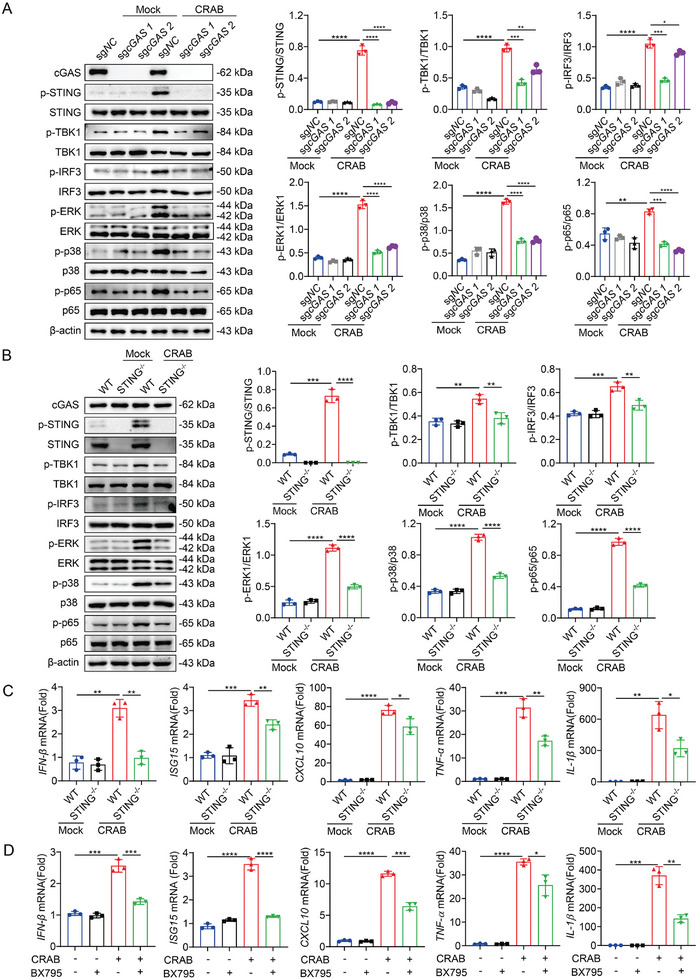
cGAS‐STING‐TBK1 contributes to CRAB‐induced type I IFN response. A) Immunoblot analysis of protein expression in cGAS knockout iBMDMs generated by CRISPR‐Cas9 and treated with CRAB for 2 h at an MOI of 10 (*n* = 3). B) Immunoblot analysis of protein expression in WT and STING^−/−^ mouse‐derived peritoneal macrophages treated with CRAB for 2 h at an MOI of 10 (*n* = 3). C) qRT‐PCR analysis of gene expression in WT and STING^−/−^ mouse‐derived peritoneal macrophages treated with CRAB for 4 h at an MOI of 10 (*n* = 3). D) qRT‐PCR analysis of gene expression in peritoneal macrophages untreated or pretreated with BX795 (2 µm) for 2 h and infected with CRAB for 4 h at an MOI of 10 (*n* = 3). The data are expressed as the mean ± SD; *p < 0.05, **p < 0.01, ***p < 0.001, ****p < 0.0001.

CRISPR‐Cas9 gene editing was used to create a complete deletion of cGAS in iBMDMs. As shown in **Figure** **4**A, cGAS deficiency significantly reduced the phosphorylation of STING, TBK1, IRF3, NF‐κB p65, p38, and ERK induced by CRAB infection. We next confirm this finding in macrophages derived from STING^−/−^ mice. Consistent with the results observed in the cGAS^−/−^ iBMDMs, STING deficiency suppressed the phosphorylation of STING, TBK1, IRF3, NF‐κB p65, p38, and ERK induced by CRAB infection, and inhibited the up‐regulation of type I IFN (IFN‐β, ISG15, and CXCL10) and pro‐inflammatory cytokines (TNF‐α and IL‐1β) induced by CRAB infection (Figure 4B,C). BX975, a specific inhibitor of TBK1, was used to verify these findings.^[^ ^22^ ^]^ After BX975 treatment, the up‐regulation of IFN‐β, ISG15, CXCL10, TNF‐α, and IL‐1β induced by CRAB infection was significantly decreased in macrophages (Figure 4D; Figure S3, Supporting Information). Collectively, these results demonstrated that CRAB infection elicited a robust type I IFN response through the cGAS‐STING signaling pathway.

### 2.4 CRAB Triggers mtDNA Release to Induce cGAS‐STING Pathway Activation

**Figure 5:**
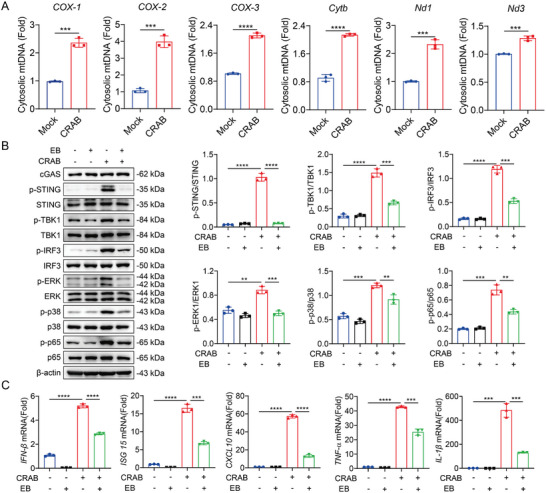
CRAB triggers mtDNA release to induce cGAS‐STING pathway activation. A) qRT‐PCR analysis of mtDNA in the cytosol of peritoneal macrophages infected with CRAB for 4 h at an MOI of 10 (*n* = 3). B) Immunoblot analysis of protein expression in EB‐treated and untreated peritoneal macrophages infected with CRAB for 2 h at an MOI of 10 (*n* = 3). C) qRT‐PCR analysis of gene expression in untreated and EB‐treated peritoneal macrophages infected with CRAB for 4 h at an MOI of 10 (*n* = 3). The data are expressed as the mean ± SD; *p < 0.05, **p < 0.01, ***p < 0.001, ****p < 0.0001.

To elucidate the mechanism of cGAS‐STING pathway activation, we further tested the level of mtDNA in the cytoplasm, which is the critical initial factor for triggering cGAS‐STING.^[^ ^23^ ^]^ CRAB infection increased the level of mitochondrial genes in the cytosolic fractions, indicating that CRAB infection resulted in the release of mtDNA to cytoplasm (**Figure** **5**A). To clarify the relationship between cGAS‐STING pathway activation and mtDNA leakage into the cell cytosol during CRAB infection, we depleted the mtDNA using ethidium bromide (EB) in mouse macrophages.^[^ ^24^ ^]^ mtDNA depletion suppressed the phosphorylation of STING, TBK1, IRF3, NF‐κB p65, p38, and ERK induced by CRAB infection, and inhibited the up‐regulation of type I IFN (IFN‐β, ISG15, and CXCL10) and pro‐inflammatory cytokines (TNF‐α and IL‐1β) induced by CRAB infection (Figure 5B,C). These results indicated that CRAB infection induced the release of mtDNA into the cytosol to activate the cGAS‐STING pathway, and mtDNA acted as a danger signal to induce a type I IFN response.

### 2.5 Mitochondrial Dysfunction Mediates mtDNA Release and cGAS‐STING Pathway Activation During CRAB Infection

**Figure 6:**
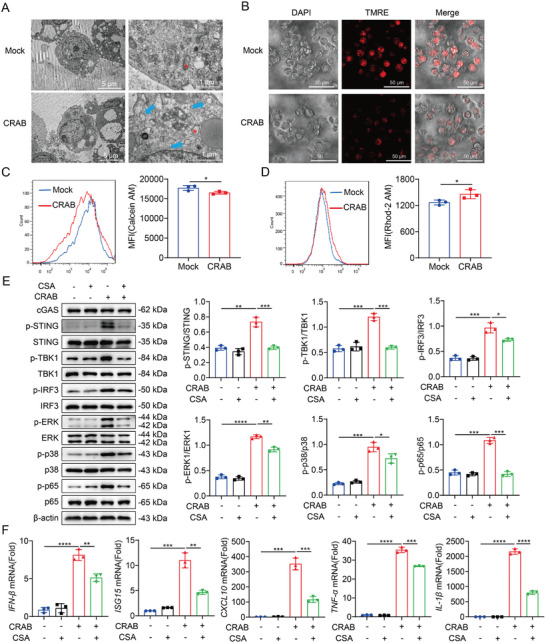
Mitochondrial dysfunction mediates mtDNA release and cGAS‐STING pathway activation during CRAB infection. A) Transmission electron microscopy images of peritoneal macrophages infected with CRAB for 4 h at an MOI of 10. Scale bar: 5 µm and 1 µm. B) Representative fluorescence micrographs of mitochondrial membrane potential by assessing TMRE in peritoneal macrophages uninfected or infected with CRAB for 4 h at an MOI of 10. Scale bar: 50 µm. The mean fluorescence index (MFI) of the mPTP level C) and the mitochondrial Ca^2+^ concentration D) in peritoneal macrophages uninfected or infected with CRAB for 4 h at an MOI of 10 were measured by flow cytometry (*n* = 3). E) Immunoblot analysis of protein expression in untreated or CsA‐treated peritoneal macrophages infected with CRAB for 2 h at an MOI of 10 (*n* = 3). F) qRT‐PCR analysis of gene expression in untreated and CsA‐treated peritoneal macrophages infected with CRAB for 4 h at an MOI of 10 (*n* = 3). The data are expressed as the mean ± SD; *p < 0.05, **p < 0.01, ***p < 0.001, ****p < 0.0001.

Increased mitochondrial permeability mediated by the mPTP leads to the release of mtDNA.^[^ ^25^ ^]^ To further explore how CRAB induces mtDNA release into the cytoplasm, morphological changes in mitochondria were determined by transmission electron microscopy (TEM). CRAB induced the loss of mitochondrial cristae and swelling of mitochondria in macrophages (**Figure** **6**A). Furthermore, macrophages infected with CRAB showed hyperpolarization of the mitochondrial membrane potential (shown by TMRE), an increase in mPTP levels (shown by calcein AM) and increased mitochondrial Ca^2+^ levels (shown by Rhod‐2 AM) (Figure 6B–D). Collectively, these results indicated that CRAB infection induced mitochondrial dysfunction and mtDNA release. To confirm the role of the mPTP in the activation of the cGAS‐STING pathway, the mPTP inhibitor cyclosporin A (CsA) was used to block the mPTP opening.^[^ ^26^ ^]^ CsA reversed CRAB‐induced mPTP opening. Furthermore, CsA treatment decreased the phosphorylation of STING, TBK1, IRF3, NF‐κB p65, p38, and ERK induced by CRAB infection, inhibited the up‐regulation of type I IFN (IFN‐β, ISG15, and CXCL10) and pro‐inflammatory cytokines (TNF‐α and IL‐1β) induced by CRAB infection (Figure 6E,F). Taken together, our results demonstrated that cGAS‐STING pathway activation in CRAB infection was initiated by mPTP opening‐mediated mtDNA leakage.

### 2.6 CRAB Delivers OMP38 via OMVs into Mitochondria to Activate the cGAS‐STING Pathway

**Figure 7:**
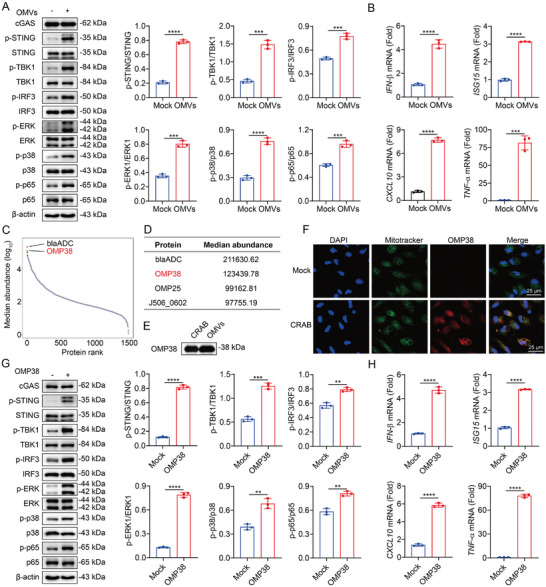
CRAB delivers OMP38 via OMVs into mitochondria to activate the cGAS‐STING pathway. A) Immunoblot analysis of protein expression in untreated or OMVs (20 µg mL^−1^)‐treated peritoneal macrophages for 2 h (*n* = 3). (B) qRT‐PCR analysis of gene expression in untreated or OMVs (20 µg mL^−1^)‐treated peritoneal macrophages for 4 h (*n* = 3). C) Ranked protein abundances in CRAB‐derived OMVs. D) Top 4 proteins based on rank abundance. E) Immunoblot analysis of protein expression in CRAB and CRAB‐derived OMVs. F) Representative fluorescence micrographs of peritoneal macrophages untreated or OMVs for 1 h and stained with DAPI (blue), MitoTracker (green), and OMP38 (red). Scale bar: 25 µm. G) Immunoblot analysis of protein expression in untreated or OMP38 (10 µg mL^−1^)‐treated peritoneal macrophages for 2 h (*n* = 3). H) qRT‐PCR analysis of gene expression in untreated or OMP38 (10 µg mL^−1^)‐treated peritoneal macrophages for 4 h (*n* = 3). The data are expressed as the mean ± SD; *p < 0.05, **p < 0.01, ***p < 0.001, ****p < 0.0001.

Pathogenic bacteria induce mitochondrial dysfunction by delivering toxins via OMVs.^[^ ^27^ ^]^ The OMVs were isolated from CRAB and characterized using transmission electron microscopy (TEM) and nanoparticle tracking analysis (NTA) (Figure S4, Supporting Information). To elucidate the mechanism of CRAB‐induced mitochondrial dysfunction, OMVs from CRAB were isolated and the phosphorylation of STING, TBK1, IRF3, NF‐κB p65, p38, and ERK and type I IFN (IFN‐β, ISG15, and CXCL10) and pro‐inflammatory cytokines (TNF‐α) were upregulated in OMVs treated‐macrophages (**Figure** **7**A,B). Mass spectrometry‐based high‐throughput proteomics analysis of CRAB‐derived OMVs showed that OMP38 is the most enriched virulence factor (Figure 7C,D). OMP38 was high expressed in CRAB‐derived OMVs and localized to the mitochondria during CRAB infection (Figure 7E,F). Recombinant *A. baumannii* OMP38 treatment upregulated the phosphorylation of STING, TBK1, IRF3, NF‐κB p65, p38 and ERK, upregulated type I IFN (IFN‐β, ISG15, and CXCL10) and pro‐inflammatory cytokines (TNF‐α) (Figure 7G,H). These results indicated that CRAB delivered OMP38 to mitochondria via OMVs to induce the cGAS‐STING pathway activation.

### 2.7 EGCG Blocks Activation of the cGAS‐STING Pathway and Ameliorates CRAB‐Induced Inflammatory Lung Injury

**Figure 8:**
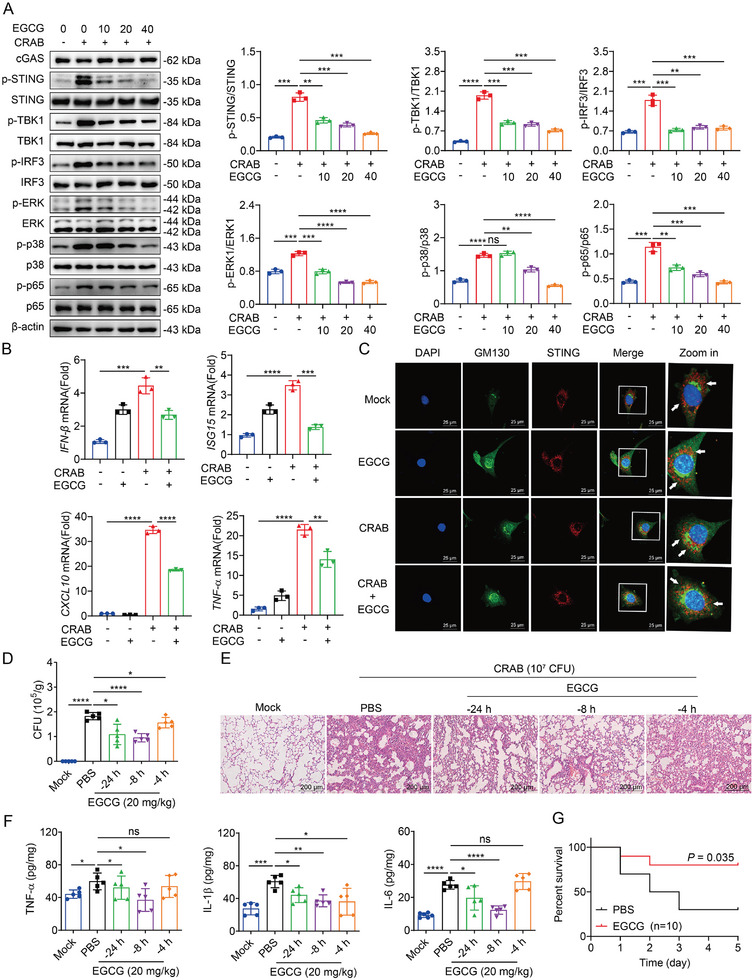
EGCG blocks activation of the cGAS‐STING pathway and ameliorates CRAB‐induced inflammatory lung injury. A) Immunoblot analysis of protein expression in peritoneal macrophages pretreated with EGCG (10, 20, 40 µg mL^−1^) and infected with CRAB for 2 h at an MOI of 10 (*n* = 3). B) qRT‐PCR analysis of gene expression in peritoneal macrophages pretreated with EGCG (10, 20, 40 µg mL^−1^) and infected with CRAB for 4 h at an MOI of 10 (*n* = 3). C) Immunofluorescence staining analysis of the Golgi apparatus translocation of STING in peritoneal macrophages pretreated with EGCG (40 µg mL^−1^) and treated with CRAB for 1 h at an MOI of 10. Scale bar: 25 µm. C57BL6 mice were pretreated with EGCG for 4, 8, or 24 h and then left uninfected or infected with 1 × 10^7^ CFU of CRAB for 24 h (*n* = 5). D) Homogenates of the lung tissues were plated to determine the bacterial CFUs per gram. E) Pathological histological analysis of lung sections stained with H&E. Scale bar: 250 µm. F) ELISA analysis of TNF‐α, IL‐1β, and IL‐6 cytokine levels in lung tissues (*n* = 5). G) C57BL6 mice were pretreated with EGCG for 8 h and then left uninfected or infected with 1 × 10^8^ CFU of CRAB (*n* = 10). The survival rate of the mice was monitored for up to 5 days. The data are expressed as the mean ± SD; *p < 0.05, **p < 0.01, ***p < 0.001, ****p < 0.0001.

To further explore the role of the cGAS‐STING pathway in CRAB infection, we screened three cGAS inhibitors: EGCG,^[^ ^28^ ^]^ G140, and RU.521.^[^ ^29^ ^]^ The results showed that EGCG, G140, and RU.521 could all inhibit the phosphorylation levels of STING, TBK1, IRF3, NF‐κB p65, p38, and ERK induced by CRAB infection (**Figure** **8**A; Figure S5A,B, Supporting Information). Notably, at comparable molar concentrations, EGCG exhibited a more pronounced inhibitory effect. Additionally, inhibition of all three cGAS inhibitors suppressed CRAB‐induced inflammatory cytokine expression, with EGCG showed superior safety (Figure 8B; Figure S5C–E, Supporting Information). Furthermore, EGCG suppressed CRAB‐induced STING translocation to the Golgi apparatus (Figure 8C). To test whether EGCG was effective in restraining the activation of the cGAS‐STING pathway in vivo, mice were intraperitoneally injected with EGCG (40 mg kg^−1^) 24 h, 8 h, or 4 h before the intranasal injection of 10^7^ CFU of CRAB. Treatment with EGCG robustly suppressed bacterial load, pathological injury, and inflammatory cytokines (TNF‐α, IL‐1β, and IL‐6) (Figure 8D–F). In addition, EGCG was intraperitoneally injected (40 mg kg^−1^) 8 h before the intranasal instillation of 10^8^ CFU of CRAB, which significantly decreased lethality (Figure 8G). Taken together, the results indicated that EGCG was effective in treating CRAB infection by blocking activation of the cGAS‐STING pathway.

## 3 Conclusion 

An alarming increase in antibiotic resistance in *A. baumannii* has been shown over the last 50 years, making effective therapies more difficult to implement and necessitating the development of new strategies.^[^ ^30^ ^]^ *A. baumannii* clearance and infection resolution depend not only on the effect of antibiotic drugs but also on the host immune response.^[^ ^31^ ^]^ To explore the role of the early innate immune system response to CRAB, mice were infected with CRAB for 6 h, and lung tissues were subjected to transcriptomic analysis by high‐throughput RNA sequencing. The results showed that CRAB activated the cGAS‐STING pathway and induced IFN‐β in lung tissue and macrophages. However, the details of the role of the early innate immune response in CRAB infection remain unknown. In this study, we demonstrated that the cGAS‐STING pathway was critically involved in CRAB infection and that STING deficiency alleviated CRAB‐induced lung injury in mice.

Alveolar macrophages (AMs) are the first line of defence of innate immune cells in the distal respiratory tract that are capable of efficient phagocytosis and killing of *A. baumannii* to limit initial replication and secrete proinflammatory cytokines and chemokines for the rapid recruitment of other innate immune cells.^[^ ^32^ ^]^ In an in vitro macrophage CRAB infection model, CRAB induced STING translocation to the Golgi apparatus to activate the cGAS‐STING pathway and upregulated the levels of p‐TBK1, p‐IRF3, p‐p65, p‐p38 and p‐ERK. cGAS or STING deficiency or TBK1 inhibition significantly decreased the activation of the cGAS‐STING pathway and the IFN response. These results further showed that CRAB infection elicits a robust IFN‐I response through the cGAS‐STING pathway. It is important to note that previous studies have demonstrated that *A. baumannii* can induce type I IFN production through a TRIF‐dependent manner.^[^ ^33^ ^]^ However, our study reveals a distinct pathway for type I IFN induction. On the other hand, the cGAS‐STING pathway influences the phosphorylation of p65, p38, and ERK and the expression of inflammatory cytokines (TNF‐α, IL‐1β and IL‐6), indicating that NF‐κB (p65), MAPK (p38) and ERK participate in the cGAS‐STING pathway.

Mitochondrial DNA within the cytosol is one of the factors that activates the cGAS‐STING pathway.^[^ ^34^ ^]^ The internalized bacterial endotoxin lipopolysaccharide (LPS) induces mtDNA release into the cytosol by activating gasdermin D to form mitochondrial pores.^[^ ^35^ ^]^ CRAB induced mitochondrial DNA translocation into the cytosol and activated the cGAS‐STING pathway, which was alleviated by the inhibition of mtDNA translocation into the cytosol. However, we failed to verify the binding of mtDNA to cGAS by immunoprecipitation or immunofluorescence staining, making it difficult to directly visualize the interaction between mtDNA and cGAS; further investigations are needed.

Mitochondrial homeostasis, including mitochondrial ultrastructure, mitochondrial reactive oxygen species (mtROS), alterations in oxidative metabolism, the tricarboxylic acid (TCA) cycle, and membrane potential, is vital for macrophage activation^[^ ^36^ ^]^ and the macrophage response to bacterial pathogens.^[^ ^37^ ^]^ CRAB altered the mitochondrial morphology and the mitochondrial calcium levels and depolarized the mitochondrial membrane potential and mPTP opening. Therefore, we further explored the contribution of the mPTP to the promotion of mtDNA release and activation of the cGAS‐STING pathway. mPTP opening during CRAB infection and inhibition of mPTP opening downregulated the cGAS‐STING pathway. These results indicated that CRAB induced mtDNA leakage through the mPTP.

OMVs are critical in facilitating host‐pathogen interactions during bacterial infection and deliver virulence factors to distant sites.^[^ ^38^ ^]^ To investigate the mechanism by which CRAB induces mitochondrial dysfunction, CRAB‐derived OMVs were isolated. The results showed that CRAB‐derived OMVs can strongly activate the cGAS‐STING pathway. OMP38 is the most abundant *A. baumannii* outer membrane protein and one of the most well‐characterized virulence factors, our results demonstrated that CRAB‐derived OMVs contain high expressed OMP38, which localized to the mitochondria during CRAB infection. Based on these results, we further found that recombinant *A. baumannii* OMP38 induced the cGAS‐STING pathway activation.

By defining the role of the cGAS‐STING pathway in CRAB infection, we further explored how to apply this finding for the clinical treatment of CRAB infection. Our results showed that inhibition of the cGAS‐STING pathway using EGCG, G140, and RU.521 effectively suppressed CRAB‐induced inflammation and EGCG showed the strongest inhibitory effect and superior safety profile. In vivo, EGCG treatment significantly decreased bacterial load, pathological damage, and inflammatory cytokines. These results suggest that targeting the cGAS‐STING pathway with EGCG could be developed as adjunctive therapies to mitigate the harmful inflammatory responses associated with CRAB infection.

In summary, we demonstrated that CRAB infection induced mitochondrial dysfunction and mPTP opening to release mtDNA into the cytosol. cGAS sensed cytoplasmic mtDNA to induce STING translocation and induced the phosphorylation of TBK1 and IFN‐β and the expression of inflammatory cytokines. Moreover, EGCG effectively suppressed cGAS activation to ameliorate CRAB‐induced inflammatory lung injury. Our findings suggest that targeting the cGAS‐STING pathway can potentially be used to treat CRAB infection and effectively prevent the development of drug resistance.

## 4 Experimental Section

### Mice

C57BL/6 mice were purchased from Shanghai Slac Animal Inc. (Shanghai, China), and STING^−/−^ mice were purchased from the Model Animal Research Center of Nanjing University. Female (18–20 g) WT or STING^−/‐^ mice were used for the experiments. All mice were housed in a specific pathogen‐free facility with a standard environment. All mouse experimental procedures were performed in accordance with the Institutional Animal Care and Use Committee of Soochow University, and all research protocols were approved by the Animal Ethical Committee of Soochow University.

### CRAB Infection

CRAB was purchased from Biobw Co., Ltd. (Beijing, China) and cultured in LB overnight at 37 °C. The following day, the bacteria were pelleted by centrifugation at 4000 × g and washed with sterile PBS three times. For RNA sequencing of lung tissue, each mouse was intranasally infected with 1 × 10^7^ CFU of CRAB for 6 h, and lung tissue was collected in TRIzol. For detection of the bacterial load in the lung tissue, the mice were intranasally infected with 1 × 10^7^ CFU of CRAB for 24 h, and the lung tissue was weighed, homogenized in PBS, serially diluted, and plated on LB plates. For survival assays, mice were intranasally infected with 1 × 10^8^ CFU of CRAB, and daily survival was recorded for 5 days to calculate the survival rate of the different groups.

### Cell Culture

Peritoneal macrophages were isolated from WT C57BL/6 and STING^−/−^ mice.^[^ ^39^ ^]^ Mice were intraperitoneally injected with sterile thioglycollate medium (4%, 2 mL). At 4 days post‐injection, the peritoneal macrophages were collected by flushing with RPMI 1640 medium. The iBMDMs were cultured in Dulbecco's modified Eagle's medium supplemented with 10% FCS and 2 mM glutamine. The cells were maintained at 37 °C in a humidified atmosphere of 5% CO_2_.

### RNA‐Sequencing Analysis

Whole‐transcriptome sequencing was performed at MetWare Biotechnology (Wuhan, China). C57BL/6 mice were infected with 1 × 10^7^ CFU of CRAB for 6 h and total RNA was extracted from lung tissues, The mRNA was fragmented with fragmentation buffer, and short RNA fragments were used as templates for synthesizing the first strand of cDNA with random hexamer primers. The second strand of cDNA was then synthesized using buffer, dNTPs (dTTP, dATP, dGTP, and dCTP), and DNA polymerase I. The double‐stranded cDNA was purified with DNA purification beads, followed by end‐repair, A‐tailing, and ligation with sequencing adapters. After selecting fragments of the appropriate size using DNA purification beads, the final cDNA library was enriched via PCR. Sequencing steps were performed following RNA‐seq protocols on the Illumina HiSeq platform. Data analysis was carried out using the Ensembl database, referencing the genome annotated as Mus_musculus. GRCm39.109. Each group consisted of three independent samples, with tissue collected from three separate mice.

### ELISA

Mouse IFN‐β production was assessed with a Mouse IFN‐β ELISA Kit (MULTI SCIENCE, #EK2236), mouse cGAMP was assessed with a cGAMP ELISA Kit (Cayman, #501700), and inflammatory cytokines were assessed via a Mouse TNF‐α ELISA Kit (Invitrogen, #88‐7324‐22), Mouse IL‐1β ELISA Kit (Invitrogen, #88‐7013‐88) and Mouse IL‐6 ELISA Kit (Invitrogen, # 88‐7064‐88) according to the manufacturers’ instructions.

### Western Blot Analysis

The cells were lysed for resolving by SDS‐PAGE and transferred onto PVDF membranes (Millipore). The membrane was blocked with 5% BSA for 2 h and incubated with primary antibodies at 4 °C overnight. The membrane was washed three times in TBST buffer (50 mm Tris, 150 mm NaCl, 0.05% Tween 20, pH 7.4) and incubated with HRP‐conjugated secondary antibodies for 1.5 h at room temperature, after which the signal was detected by using an enhanced chemiluminescence (ECL) kit (Invitrogen) following the manufacturer's specific protocol.

### RNA Extraction and qRT‐PCR Analysis

Total RNA from macrophages was extracted with TRIzol lysis reagent (A&G) according to the manufacturer's instructions, and the concentration and quality of the RNA were determined with a Nandrop One (Thermo Fisher Scientific). Reverse transcription of RNA to cDNA and qRT‒PCR were carried out. Relative expression levels were calculated by the 2^−ΔΔCT method^.

### Cytosolic DNA Isolation

Cytosolic DNA was extracted as previously described.^[^ ^24^ ^]^ Peritoneal macrophages were lysed in 500 µL of digitonin buffer (150 mm NaCl, 50 mm HEPES (pH 7.4), 40 µg mL^−1^ digitonin, protease, and phosphatase inhibitors) on a shaker for 10 min and centrifuged at 2000 × g for 10 min at 4 °C. The supernatants were transferred to new fresh tubes and centrifuged at 20 000 × g for 20 min at 4 °C. The supernatants were the cytosolic fractions, and the remained pellets were nuclear fractions and DNA was extracted using a DNA isolation kit (Tiangen). Real‐time PCR was used to quantify the mitochondrial DNA, and the RPS18 (ribosomal protein S18) was quantified for normalization.

### Immunofluorescence Staining

Macrophages seeded in glass‐bottom cell culture dishes were fixed in 4% paraformaldehyde and permeabilized with 0.1% Triton X‐100 for 10 min. After being blocked with 5% bovine serum albumin (BSA) for 1 h, the cells were incubated with specific primary antibodies overnight at 4 °C. The cells were then incubated with appropriate Alexa Fluor 647/FITC‐conjugated secondary antibodies for 1 h at room temperature. The cell nuclei were counterstained with DAPI. Images were obtained with a Leica TCS SP8 confocal microscope.

### Generation of TLR4 and cGAS Knockout Cell Lines

The lentiCRISPR V2 plasmid was used to construct TLR4 and cGAS KO cells, and the following RNA sequences were used: TLR4 sg1: 5′‐ TAATATTACCTACCAATGCA‐3′; TLR4 sg2: 5′‐ TGAGTTTCTGATCCAT‐GCAT’; cGAS sg1: 5′‐CACCGTGTTTAAACTGGAAGTCCCC‐3′; cGAS sg2: 5′‐CACCGATTCTTGTAGCTC‐AATCCTG‐3′. The TLR4 and cGAS lentiCRISPR V2 plasmid and packaging plasmids pVSVg and psPAX2 were transfected into HEK293T cells. The lentiviruses were collected, and TLR4 and cGAS KO cells were selected with 2.5 µg mL^−1^ puromycin for 7 days.

### Transmission Electron Microscopy

The macrophages were immobilized in a solution containing 2.5% glutaraldehyde and 150 mm sodium cacodylate (pH 7.4) overnight at 4 °C. Next, the cells were fixed with 1% OsO_4_, stained with uranyl acetate, dehydrated with ethanol, and ultimately encased in epoxy resin. The sections were then treated with uranyl acetate and placed on Formvar‐coated grids. The visual representations were captured by a Hitachi HT‐7800 transmission electron microscope.

### Isolation of OMVs

The OMVs were isolated from CRAB culture supernatants according to previous studies.^[^ ^40^ ^]^ The bacterial culture supernatants were collected and filtered through a 0.22‐µm sterile filter. After filtration, supernatants were further ultracentrifuged at 150 000 × g for 2 h at 4 °C using a 70 Ti rotor (Beckman Coulter, USA). The OMVs were collected and resuspended in phosphate‐buffered saline.

### Characteristics of OMVs

For morphological analysis, OMVs were placed on a copper grid for 5 min, stained with 2% uranyl acetate, and visualized using transmission electron microscopy (TEM) (Hitachi, Japan). For nanoparticle tracking analysis, the particle concentration and size distribution of OMVs were measured using a Zeta‐View Particle Metrix system (Particle Metrix, Germany).

### Statistical Analysis

Statistical analysis of the experimental data was performed by using GraphPad Prism 8 (GraphPad Software, California, USA). Statistically significant differences were determined by unpaired two‐tailed Student's *t*‐tests. *p* values were determined by the log‐rank (Mantel–Cox) test and indicated differences in survival rates between mice from different groups. The error bars represent ± SD.

## Conflict of Interest

The authors have no conflicts of interest.

## Ethics Approval

All animal experimental procedures received ethical approval from the Ethics Committee of the First Affiliated Hospital of Soochow University (approval no. 2023–462).

## Author Contributions

Y.Y. performed the experiments and edited the manuscript. Y.Y.Z., J.J.Z., J.J.L., and L.G. participated in the data collection and analysis. L.W. was involved in the investigation and writing. Z.Y.L. and J.A.H. were responsible for funding acquisition and finalizing the manuscript. Correspondence and requests for materials should be addressed to Z.L.

## Supporting information



Supporting Information

## Data Availability

The data that support the findings of this study are available from the corresponding author upon reasonable request.
