# Supplementary material for: OMP38 of Carbapenem‐Resistant Acinetobacter Baumannii‐Mediated mtDNA Release Activates the cGAS‐STING Signaling to Induce Inflammatory Response
Source: Adv Sci (Weinh). 2024 Dec 4;12(4):2408292. doi: 10.1002/advs.202408292 (PMC11775518; doi:10.1002/advs.202408292)
Supplement: Supplementary file 1 — Supporting Information [file ADVS-12-2408292-s001.docx]

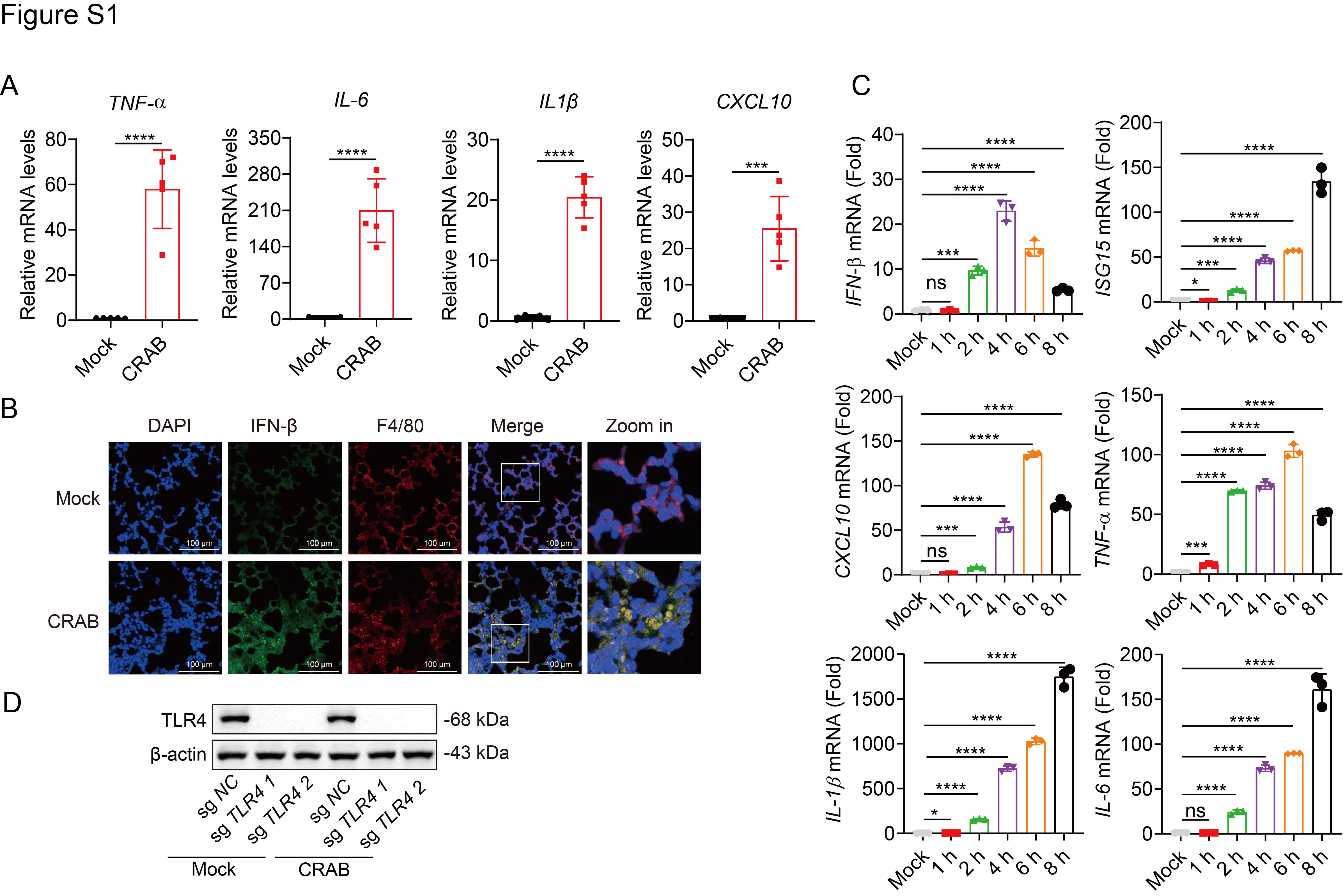


**Figure S1. CRAB infection induces type I IFN response associated genes expression.** (A) qRT-PCR analysis of gene expression in lung tissues of uninfected mice or mice infected with 1×10^7^ CFU CRAB for 6 h (*n* = 5). (B) Immunofluorescence staining analysis of the expression of IFN-β in macrophages in lung tissues of uninfected mice or mice infected with 1×10^7^ CFU CRAB for 6 h . (C) qRT-PCR analysis of gene expression in mouse peritoneal macrophages uninfected or infected with CRAB for 1, 2, 4, 6, or 8 h at an MOI of 10 (*n* = 3). (D) Immunoblot analysis of protein expression in TLR4 knockout iBMDMs generated by CRISPR-Cas9 and treated with CRAB for 2 h at an MOI of 10. The data are expressed as the mean ± SD; **P*＜0.05, ***P*＜0.01, ****P*＜0.001, *****P*＜0.0001.


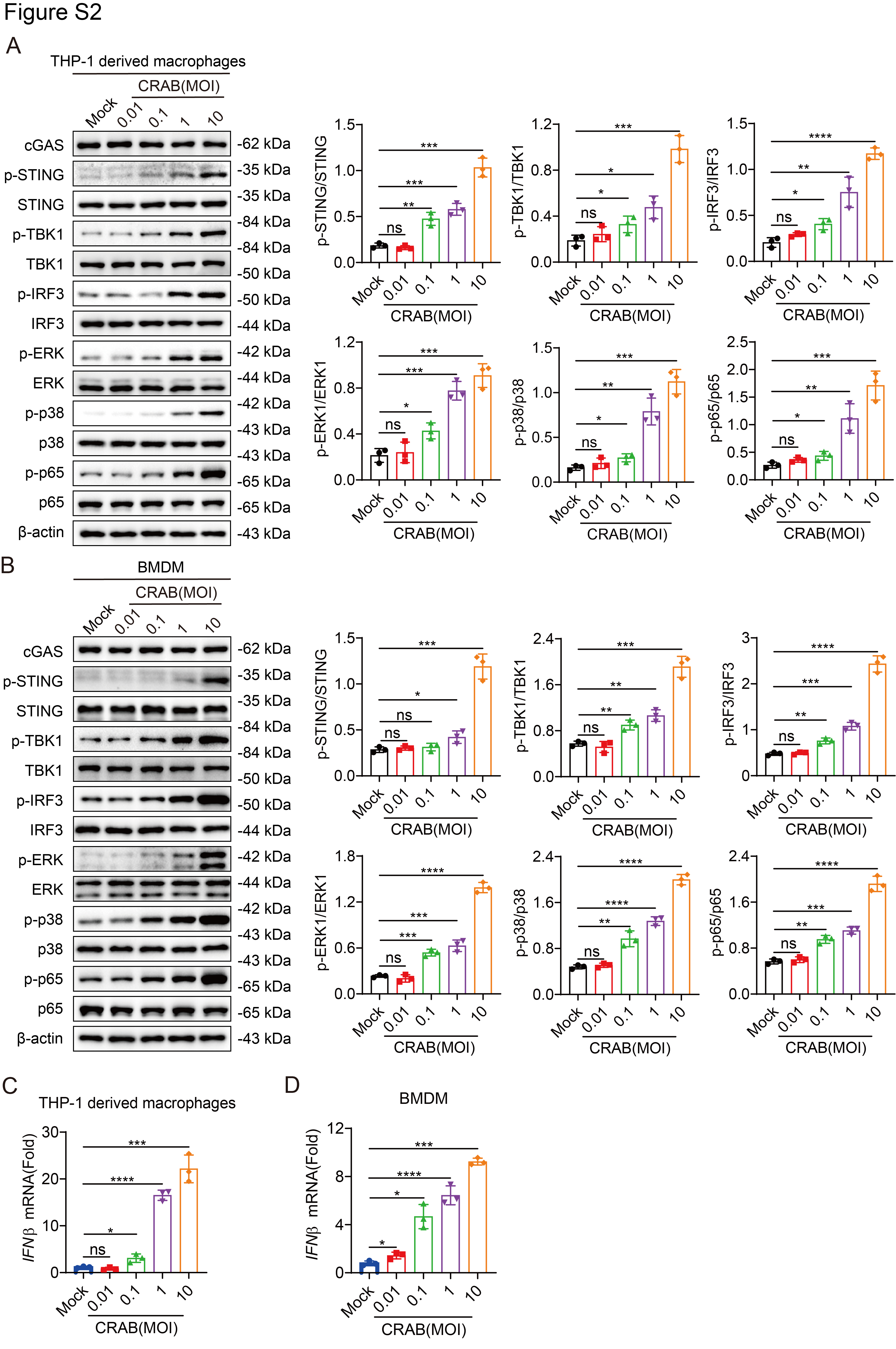


**Figure S2. The CRAB-induced activation of the cGAS-STING pathway in THP-1 derived macrophages and BMDMs.** (A) Immunoblot analysis of protein expression in THP-1 derived macrophages infected with CRAB for 2 h at MOI of 0.01, 0.1, 1, and 10. (B) Immunoblot analysis of protein expression in BMDMs infected with CRAB for 2 h at an MOI of 10. (C) qRT-PCR analysis of IFN-β in THP-1 derived macrophages infected with CRAB for 4 h at MOI of 0.01, 0.1, 1, and 10. (D) qRT-PCR analysis of IFN-β in BMDMs infected with CRAB for 4 h at MOI of 0.01, 0.1, 1, and 10. The data are expressed as the mean ± SD; **P*＜0.05, ***P*＜0.01, ****P*＜0.001, *****P*＜0.0001.


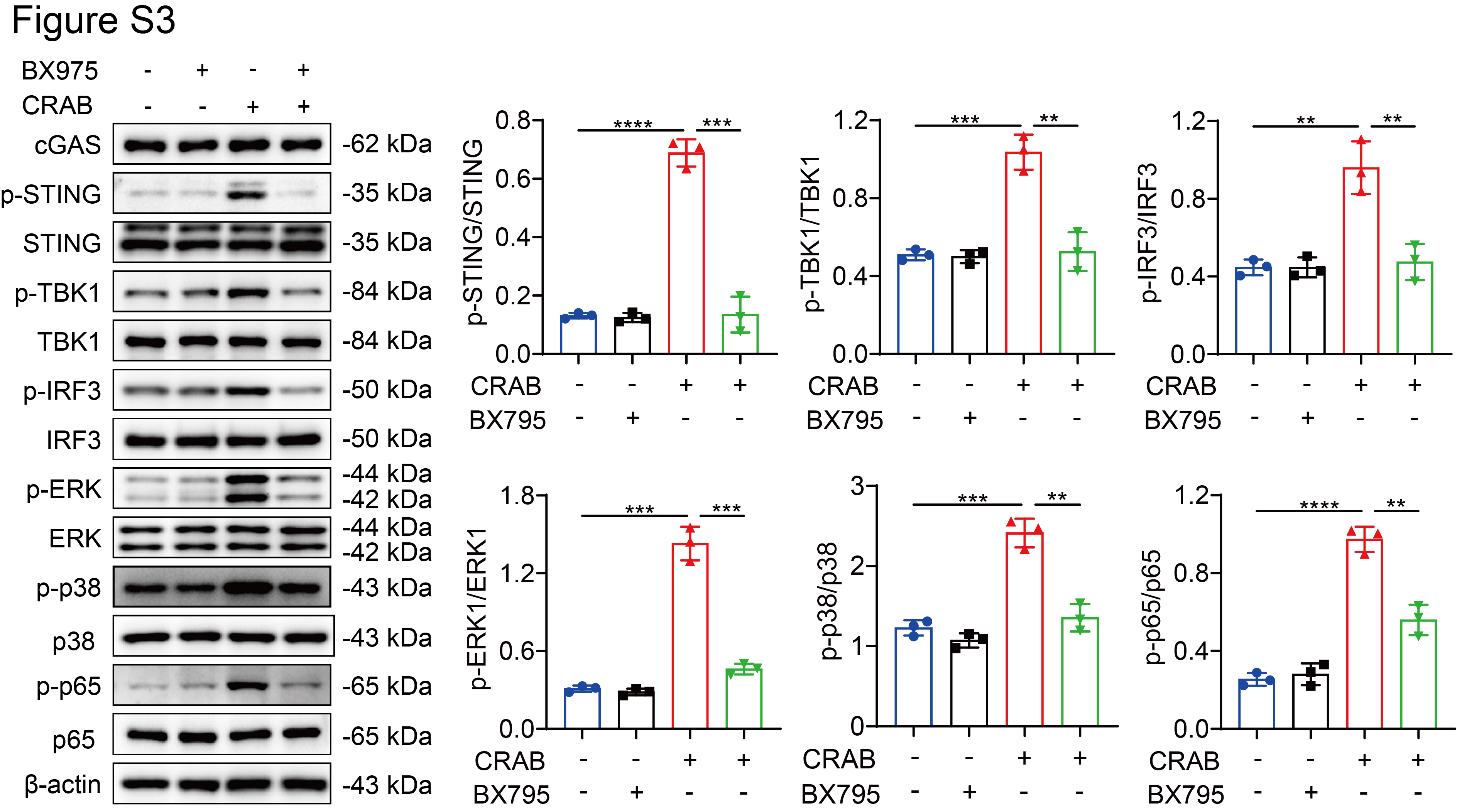


**Figure S3. CRAB infection induces type I IFN response associated genes expression.** Immunoblot analysis of protein expression in peritoneal macrophages untreated or pretreated with BX795 (2 μM) for 2 h and infected with CRAB for 2 h at an MOI of 10. The data are expressed as the mean ± SD; **P*＜0.05, ***P*＜0.01, ****P*＜0.001, *****P*＜0.0001.


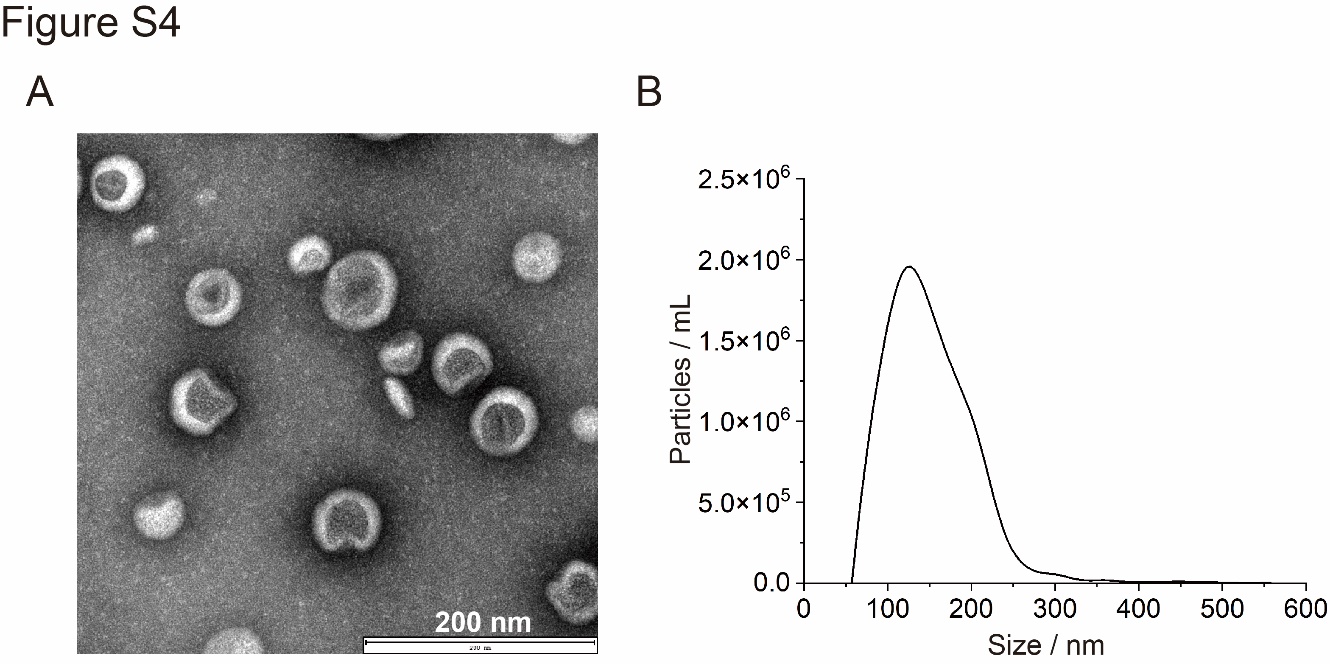


**Figure S4. CRAB derived OMVs characteristics.** (A) TEM image of OMVs and (B) Nanoparticle tracking analysis isolated from CRAB. Scale bar: 200 nm.


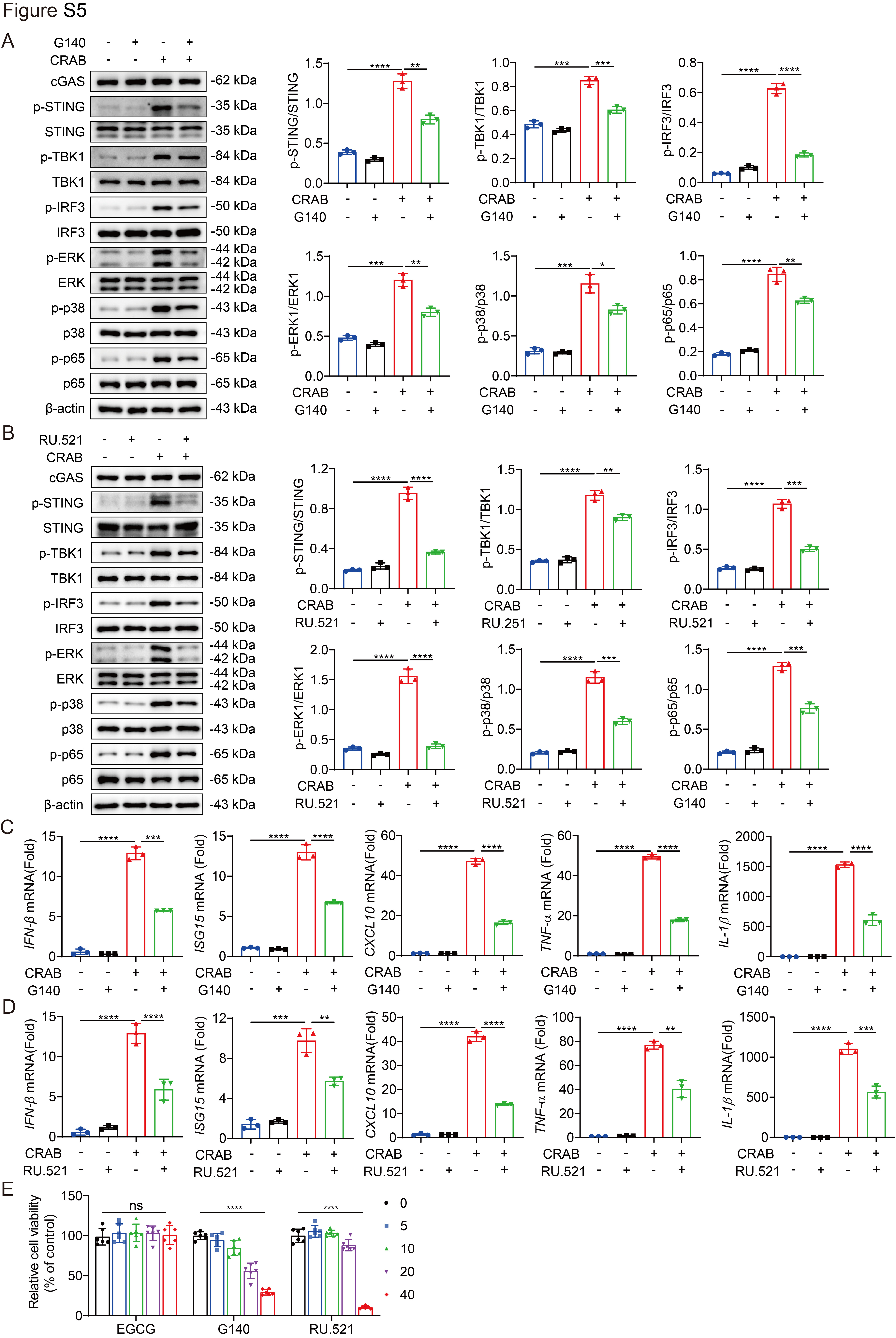


**Figure S5.** **G140 and RU.521 blocks activation of the cGAS-STING pathway.** (A) Immunoblot analysis of protein expression in peritoneal macrophages pretreated with G140 (10 μM) and infected with CRAB for 2 h at an MOI of 10 (*n* = 3). (B) Immunoblot analysis of protein expression in peritoneal macrophages pretreated with RU.521 (10 μM) and infected with CRAB for 2 h at an MOI of 10 (*n* = 3). (C) qRT-PCR analysis of genes expression in peritoneal macrophages pretreated with G140 (10 μM) and infected with CRAB for 4 h at an MOI of 10 (*n* = 3). (D) qRT-PCR analysis of genes expression in peritoneal macrophages pretreated with RU.521 (10 μM) and infected with CRAB for 4 h at an MOI of 10 (*n* = 3). (E) Cytotoxicity assay of macrophages treated with EGCG, G140, and RU.521 for 24 h (μM) (*n* = 6). The data are expressed as the mean ± SD; **P*＜0.05, ***P*＜0.01, ****P*＜0.001, *****P*＜0.0001.
